# Alginate-modifying enzymes: biological roles and biotechnological uses

**DOI:** 10.3389/fmicb.2015.00523

**Published:** 2015-05-27

**Authors:** Helga Ertesvåg

**Affiliations:** Department of Biotechnology, Norwegian University of Science and TechnologyTrondheim, Norway

**Keywords:** alginate, alginate lyase, mannuronan C-5 epimerases, alginate acetylation, alginate deacetylase

## Abstract

Alginate denotes a group of industrially important 1-4-linked biopolymers composed of the C-5-epimers β-D-mannuronic acid (M) and α-L-guluronic acid (G). The polysaccharide is manufactured from brown algae where it constitutes the main structural cell wall polymer. The physical properties of a given alginate molecule, e.g., gel-strength, water-binding capacity, viscosity and biocompatibility, are determined by polymer length, the relative amount and distribution of G residues and the acetyl content, all of which are controlled by alginate modifying enzymes. Alginate has also been isolated from some bacteria belonging to the genera *Pseudomonas* and *Azotobacter*, and bacterially synthesized alginate may be *O*-acetylated at O-2 and/or O-3. Initially, alginate is synthesized as polymannuronic acid, and some M residues are subsequently epimerized to G residues. In bacteria a mannuronan C-5-epimerase (AlgG) and an alginate acetylase (AlgX) are integral parts of the protein complex necessary for alginate polymerization and export. All alginate-producing bacteria use periplasmic alginate lyases to remove alginate molecules aberrantly released to the periplasm. Alginate lyases are also produced by organisms that utilize alginate as carbon source. Most alginate-producing organisms encode more than one mannuronan C-5 epimerase, each introducing its specific pattern of G residues. Acetylation protects against further epimerization and from most alginate lyases. An enzyme from *Pseudomonas syringae* with alginate deacetylase activity has been reported. Functional and structural studies reveal that alginate lyases and epimerases have related enzyme mechanisms and catalytic sites. Alginate lyases are now utilized as tools for alginate characterization. Secreted epimerases have been shown to function well *in vitro*, and have been engineered further in order to obtain enzymes that can provide alginates with new and desired properties for use in medical and pharmaceutical applications.

## Introduction

Alginate is defined as a linear polymer consisting of 1-4-linked mannuronic acid (M) and guluronic acid (G). The polysaccharide is currently manufactured from brown algae, and used as a viscosifier, emulsifier and gel forming agent in many different applications (Skjåk-Bræk et al., 2015). Alginate has also been found in some red algae (Usov et al., 1995) and is produced by some bacteria belonging to the genera *Azotobacter* (Gorin and Spencer, 1966) and *Pseudomonas* (Linker and Jones, 1966). Genes encoding putative alginate biosynthetic gene clusters are present in many other sequenced bacterial genomes.

**FIGURE 1:**
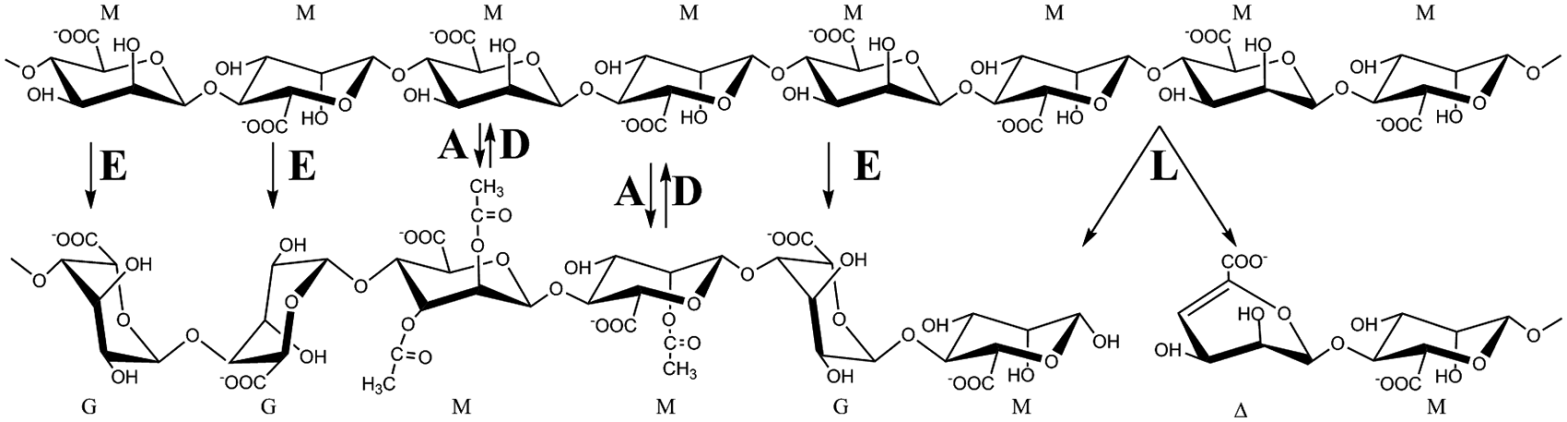
**Enzymatic modifications of alginate.** Alginate is synthesized as polymannuronan (top molecule). The mannuronic acid residues (M) may then be epimerized to guluronic acid residues (G) by mannuronan C-5 epimerases (E), O2 or O3 acetylated by mannuronan acetylases (A), or degraded by alginate lyases (L) resulting in an unsaturated 4-deoxy-L-erythro-hex-4- enepyranosyluronate residue (Δ). Acetyl groups may be removed by deacetylases (D).

Initially alginate is synthesized as mannuronan by polymerization of GDP-mannuronic acid (**Figure 1**). This homopolymer is then modified by alginate modifying enzymes. Four groups of such enzymes have been characterized; alginate acetylases, alginate deacetylases, alginate lyases, and mannuronan C-5-epimerases (**Figure 1**). As a result of the action of these alginate-modifying enzymes the term alginate denotes a family of polysaccharides with differing chemical composition and properties. In this review the relationship between the composition of a specific alginate and its physical properties are briefly described as are the uses of alginates by the alginate-producing organisms. The different enzyme groups are then addressed separately, before the review concludes by describing how alginate lyases and mannuronan C-5 epimerases may be used *in vitro.*

## Post-Polymerization Modifications Determine the Functional Properties of Alginates

Alginates are utilized industrially because they bind cations, bind water, provide viscosity to a solution, and may form gels. It has long been known that both the ratio and distribution of G-residues and the polymer length influence these properties, and it is conceptually better to consider alginate as a group of chemically related polymers with different properties. A given alginate molecule can formally be described as composed of at least one of the three possible block-types: consecutive M-residues (M-blocks), consecutive G-residues (G-blocks) and strictly alternating M and G-residues (MG-blocks; **Figure 1**). The length of these may vary from two residues and upward. G-blocks and MG-blocks may be cross-linked by divalent cations and form gels. Currently NMR is the preferred method used to characterize alginate composition, since it makes it possible to determine the frequencies of the monomers M and G, the diads MM, MG/GM, and GG, and the eight different triads (Grasdalen et al., 1981; Grasdalen, 1983).

The block types and block lengths influence the flexibility of the polymer and thus the viscosity of the solution as well as the stability, permeability, and syneresis of gels (Skjåk-Bræk et al., 2015). Studies have shown that alginates with a high M-content are immunogenic, while G-blocks does not stimulate an immune response (Espevik et al., 1993), and this has important implications when alginate capsules for implantation of cells in humans are designed. Alginate synthases produce polymannuronic acid, and the G-residues are later introduced by mannuronan C-5 epimerases. Many alginate-producing organisms even encode several C-5 epimerases enabling them to tailor the synthesized alginates to a specific use.

Bacterial alginates may be *O*-acetylated at some of the C-2 and C-3 carbons of the mannuronic acid residues, and acetylation takes place during transport through the periplasm (Baker et al., 2014). Acetyl groups affects the viscosity and flexibility of the molecule and increases its ability to bind water (Skjåk-Bræk et al., 1989b).

Polymer length is another variable important for viscosity and gel strength. Any given alginate preparation contains a mixture of molecules with different lengths, the lengths, and degree of polydispersity might be found by SEC-MALLS (Bakkevig et al., 2005). It is not known what causes the synthesis of a particular alginate molecule to terminate. During manufacturing of alginate from brown algae some depolymerization takes place, both due to physical forces, but also by alginate lyases produced by bacteria living on the seaweeds.

## Alginates Have a Protective Role in the Alginate-Producing Organisms

The cell walls of brown algae contain a network of alginate molecules where G-blocks in the molecules are cross linked by Ca^2+^. Alginate is the most abundant component of the cell wall, which also contains fucoidan, cellulose, polyphenols, and proteins (Michel et al., 2010). Polyphenols have been shown to be linked to the alginate network, thus further strengthening the cell wall (Deniaud-Bouët et al., 2014). It has been suggested that the genes involved in biosynthesis of alginate in brown algae were acquired through a horizontal gene transfer from an actinobacterium and that this enabled the formation of complex multicellular organisms within the group of Stramenopiles (Michel et al., 2010; Deniaud-Bouët et al., 2014).

Wild type strains belonging to the genus *Azotobacter* seem to produce alginate constitutively, at least under laboratory conditions. Alginate is surrounding the vegetative cells, however, alginate-negative mutants do not display impaired growth or fitness in the laboratory. Most studies addressing nitrogen fixation in *Azotobacter vinelandii* are utilizing mutants that are unable to produce alginate due to a transposon insertion in the sigma factor gene necessary for alginate production (Setubal et al., 2009). When bacteria belonging to the genus *Azotobacter* sense a lack of carbon source, the cell will enter a dormant stage where it is protected by a G-block-containing cyst coat. The cyst can survive desiccation for years, and when conditions again allow for growth the cyst will germinate (Sadoff, 1975). It has later been proven that only alginate-producing cells may produce cysts that are able to germinate after storage (Campos et al., 1996), showing that the ability to produce alginate containing G-blocks is important for the long-time fitness of these bacteria in nature.

Only a few species belonging to the genus *Pseudomonas* are able to produce alginate, and alginate production in these species are tightly regulated and only turned on in response to specific stimuli; typically those causing cell wall stress (Wood and Ohman, 2012). It has been shown that alginate participates in forming the matrix of the *P. aeruginosa* biofilm and in providing resistance toward the host immune system (Pier et al., 2001; Tielen et al., 2005; Harmsen et al., 2010). Acetylated M residues are also protected from cleavage by some alginate lyases (Skjåk-Bræk et al., 1989a) and from epimerization.

## Alginate Lyases

In nature alginate lyases are synthesized by organisms that utilize alginate as a carbon source, as well as by the alginate-producing bacteria. So far no alginate lyase produced by brown algae has been identified. Several alginate lyases have been thoroughly studied, and this has provided a detailed understanding of the catalytic mechanism of these enzymes.

### Structure-Function Studies of Alginate Lyases

As depicted in **Figure 1** an alginate molecule may contain four different bonds; M–M, M–G, G–M, and G–G, and most lyases display very different reaction rates toward the different bonds. Alginate lyases are divided into G-specific (EC4.2.2.11) and M-specific (EC4.2.2.3) enzymes. However, this classification does not distinguish between enzymes cleaving for instance either M–G or G–M. Moreover, not all alginate lyases accept acetylated substrates (Wong et al., 2000).

Polysaccharide lyases are divided into families based on sequence similarities. By the end of 2014 22 polysaccharide lyase families had been identified in the CAZY database, moreover some characterized enzymes are not yet classified in a family (Lombard et al., 2010). Alginate lyases are found in the polysaccharide lyase families PL5, PL6, PL7, PL14, PL15, PL17, and PL18, and in the group of unclassified polysaccharide lyases. The assignment to this number of families implies that alginate lyases structurally are quite diverse. The polysaccharide lyase families PL5, PL15, and PL17 have been found to have an (α/α)*_n_* toroid fold structure, PL6 has a β-helix fold structure, while PL7, PL14, and PL18 are folded as β-jelly rolls. The structural folds of the polysaccharide lyases have been reviewed recently (Garron and Cygler, 2010, 2014) and will not be further discussed here.

Most studied alginate lyases functions endolytically, i.e., they cleave the alginate molecules internally, and do not produce significant amounts of oligomers at the beginning of the reaction. If the reaction is allowed to proceed, the final products usually are dimers, trimers, tetramers, or pentamers. However, several exolyases which removes single residues from the end of the polymer have been described (Ochiai et al., 2010; Thomas et al., 2012; Park et al., 2014).

Gacesa (1987) first proposed the reaction mechanism for alginate lyases. Firstly the negative charge on the carboxylate anion is shielded by the enzyme. This allows for the proton to be abstracted from C-5. The intermediate enolate ion is proposed to be stabilized by resonance. Finally, electron transfer from carboxyl group results in the formation of a double bond between C-4 and C-5 and cleavage of the *O*-glycosidic bond. Cleavage has been found to be facilitated by an amino acid residue acting as an acid (Garron and Cygler, 2010). The new non-reducing end will contain a 4-deoxy-L-*erythro*-hex-4-enepyranosyluronate (Δ) as depicted in **Figure 1**. This double bond absorbs at 235 nm and is used to quantitate alginate lyase activity (Preiss and Ashwell, 1962a). For most alginate lyases the negative charge is stabilized by glutamine, arginine or asparagine. It is important for the catalytic mechanism that for M-residues the C-5-proton and the departing oxygen on C-4 lie *syn* relative to each other, while they lie *anti* relative to each other for G-residues (**Figure 1**). For the studied alginate lyases it has been found that for M-specific lyases the C-5 proton is abstracted by a tyrosine that also acts as the acid that facilitates the cleavage of the *O*-glycosidic bond. For lyases acting on G-residues the C-5-proton is abstracted by a histidine while a tyrosine again acts as the acid (Garron and Cygler, 2014). Alginate lyases belonging to the PL6 family do not follow this scheme. They use Ca^2+^ as neutralizer, lysine as the proton-abstracting residue, and arginine as the acid (Garron and Cygler, 2014).

### Alginate Lyases in Alginate-Producing Bacteria

**FIGURE 2:**
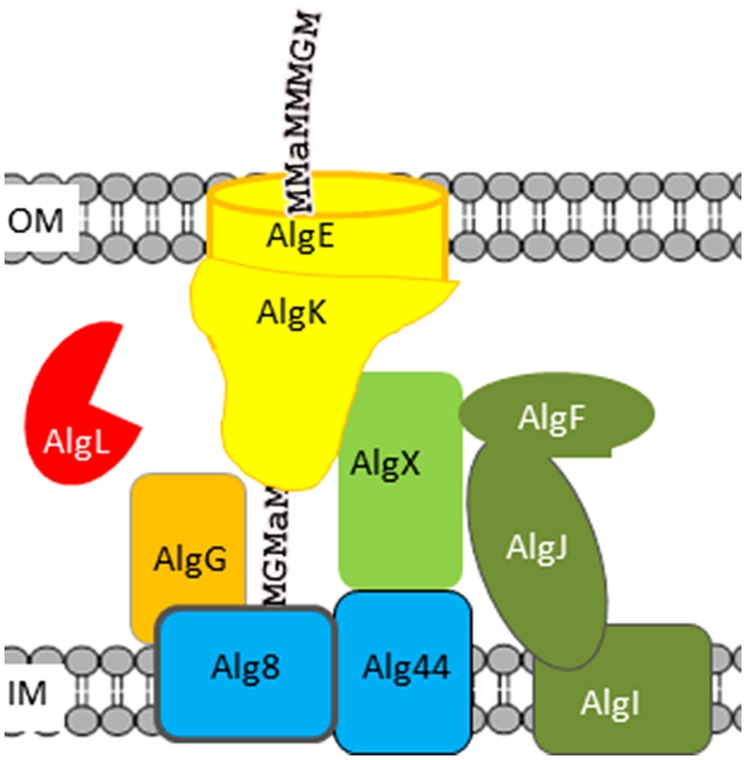
**Model of the *Pseudomonas aeruginosa* alginate biosynthetic complex.** Proteins involved in polymerization are colored blue, the alginate lyase red, the epimerase orange, and proteins involved in acetylation green. The names of proteins assumed to be part of the alginate polymerization and transport complex are written using black letters. Alginate is depicted as a text string; “a” denotes acetyl-groups.

So far no alginate-producing bacteria have been reported to use alginate as a carbon source. Still, in all known alginate-producing bacteria an alginate lyase is encoded in the gene cluster encoding the other proteins needed for alginate biosynthesis. This periplasmic alginate lyase seems to be involved in clearing the periplasm for mislocated alginate molecules originating from non-functional export complexes. Alginate is a polyanion, which attracts small cations. Free alginate molecules in the periplasm consequently result in an increased osmotic pressure that finally will cause lysis, and alginate-producing cells without this lyase do not survive (Bakkevig et al., 2005; Jain and Ohman, 2005). It has also been found that mutants that do not produce AlgK, AlgX, or AlgG do not produce alginate, but instead unsaturated oligomers, which would be the expected product of AlgL-degraded alginate (Jain and Ohman, 1998; Gimmestad et al., 2003; Jain et al., 2003; Robles-Price et al., 2004). These data is compatible with a model (**Figure 2**) in which the polymerized alginate is transported through a protein complex composed of the polymerase Alg8, the copolymerase Alg44, the periplasmic proteins AlgG, AlgX, and AlgK, and the outer membrane pore AlgE; and AlgL would only have access to alginate molecules that were aberrantly released to the periplasm because of a non-functional protein complex (Bakkevig et al., 2005).

*Pseudomonas aeruginosa* also encodes a second alginate lyase, Pa1167 (Yamasaki et al., 2004), but the biological function of this lyase is unknown. *A. vinelandii* encodes two alginate lyases, AlyA1 and AlyA2, which are homologous to Pa1167. AlyA1 was dispensable for growth in the laboratory, while AlyA2 was necessary for normal growth (Gimmestad et al., 2009). *A. vinelandii* further encodes two secreted alginate lyases, AlyA3 and AlgE7. AlyA3 contains an alginate lyase module homologous to AlyA1 and AlyA2 linked to three calcium-binding modules homologous to the R-modules of secreted mannuronan C-5 epimerases (See Mannuronan C-5 Epimerases). It has been shown that R-modules can bind to alginate, although the binding strength is dependent on the specific R-module as well as the structure of the alginate molecule (Buchinger et al., 2014). The cysts of *Azotobacter* sp. will germinate when they are placed in an environment with a suitable carbon source (Sadoff, 1975), and AlyA3 is needed to open the cyst capsule in order to allow the vegetative cell to escape and start growing (Gimmestad et al., 2009). AlgE7 is a bifunctional enzyme belonging to the family of secreted mannuronan C-5- epimerases and displays both mannuronan C-5-epimerase and alginate lyase activity (Svanem et al., 2001). In the absence of AlgE7 it is more difficult to separate alginate from the cells by centrifugation (Gimmestad et al., 2009), indicating that AlgE7 is used to detach cells from alginate. *A. vinelandii* also encodes an exolyase, AlyB, the biological function of which is unknown (Ertesvåg, unpublished data). The genome sequence of *A. chroococcum* was recently released (Robson et al., 2015). In addition to *algL*, the *A. chroococcum* genome contains four other genes homologous to *A. vinelandii* alginate lyase genes: *Achr_16810* is homologous to *A. vinelandii alyA1* and *alyA2*, *Achr_5040* is homologous to *A. vinelandii alyB* and two genes, *Achr_39560* and *Achr_39570*, are homologous to AlgE7.

### Alginate Lyases in Alginate-Utilizing Organisms

Alginate lyases are produced by many organisms that utilize alginate as a carbon source; bacteria, animals, and viruses (Wong et al., 2000). These organisms are either marine, utilizing the alginate produced by brown algae, or soil bacteria presumably utilizing alginate produced by *Azotobacter* sp. or alginate-producing *Pseudomonas* sp. Many bacteria have been found to secrete alginate lyases that degrade alginate into smaller oligomers that can be taken up by the cells. The oligosaccharides are further degraded by oligoalginate lyases and exotype alginate lyases into 4-deoxy-*l*-erythro-5-hexoseulose uronic acid, which then is converted to pyruvate and glyceraldehyde-1-phosphate (Preiss and Ashwell, 1962b). As an example the marine bacterium *Zobellia galactanivorans* contains two operons encoding several alginate lyases as well as the dehydrogenase and kinase needed for converting alginate into substrates used in the central carbon metabolism (Thomas et al., 2013).

Secretion of alginate lyases inflicts the risk that other cells might import the alginate oligomers generated by such extracellular enzymes. The soil bacterium *Sphingomonas* sp. A1 has developed an alginate up-take system whereby the polymer is imported into the cell through a dedicated ABC-transporter and then degraded by a series of intracellular alginate lyases with different substrate specificities. This unique system has been reviewed elsewhere (Hashimoto et al., 2010).

## Mannuronan C-5 Epimerases

Alginates isolated from brown algae and from bacteria belonging to the genus *Azotobacter* contain consecutive G-residues, while alginates isolated from *Pseudomonas* sp. so far only has been found to contain single G-residues. This can be related to the need brown algae and *Azotobacter* have in forming alginate gel networks that can provide strength to their cell wall and cyst coat, respectively. As long as Pseudomonads only use alginates to form a loosely connected capsule or as a minor component of their biofilms, they may not have the same need for G-blocks in their alginates. Still, some *Pseudomonads* encode a protein that is able to introduce G-blocks *in vitro* (Bjerkan et al., 2004a). Two types of mannuronan C-5-epimerases have been described; the Ca^2+^-independent AlgG-type, and the Ca^2+^-dependent AlgE-type.

### AlgG-Type Epimerases are Found in All Alginate-Producing Organisms

All alginate-producing bacteria encode a periplasmic mannuronan C-5- epimerase, AlgG, in the alginate biosynthesis gene cluster and the secreted alginate contains some G-residues. Polymannuronic acid forms fairly stiff molecules due to the diequatorial linkages, while introduction of G-residues provides equatorial-axial bonds that increase the flexibility of the chain (Smidsrød et al., 1973), and this might explain why a mannuronan-producing organism has not been identified in nature. In the laboratory mannuronan-producing strains encoding a mutated form of AlgG have been isolated (Chitnis and Ohman, 1990; Gimmestad et al., 2003).

Brown algae encode a large family of mannuronan C-5-epimerases that are related to the bacterial AlgG-epimerases (Nyvall et al., 2003; Michel et al., 2010). No algal epimerase gene have yet been reported expressed in any heterologous system, thus nothing is known about how these enzymes might differ as to which kind of alginate they are producing. It is, however, known that the G-content of alginates synthesized by brown algae differ between species, within different parts of the plant and with the environment of the individual plant. Since genes encoding mannuronan C-5-epimerases have been found to be differentially expressed in *Laminaria digitata* (Tonon et al., 2008), it seems probable that different epimerases are able to introduce distinct G-distribution patterns that confers the crosslinking and ionic binding properties needed in a given tissue and environment.

### The Secreted AlgE-Type Epimerases are Used to Produce G-Block-Containing Alginates in Bacteria

In contrast to what seems to be the case in algae the consecutive G-residues found in *A. vinelandii* alginates are not a result of its AlgG epimerase (Steigedal et al., 2008). However, as early as in 1969 a secreted, Ca^2+^-dependent mannuronan C-5 epimerase from *A. vinelandii* was described (Haug and Larsen, 1969), and this was the first example of epimerases active on a polymer. The enzyme attracted interest because it was able to increase the G-content in alginates *in vitro*. The earliest studies were not able to ascertain whether *A. vinelandii* secreted one or more different epimerases, but this was resolved when the genes encoding seven secreted epimerases (AlgE1-7) were identified, cloned and expressed in *E. coli* (Ertesvåg et al., 1999). These epimerases have been shown to introduce different G-distribution patterns and to be expressed differentially during the life cycle of the cell (Høidal et al., 2000). *A. vinelandii* also encodes another protein, AlgY, which is homologous to the AlgE-epimerases, but does not display any activity on mannuronan. These early studies on the secreted epimerases have been extensively reviewed earlier (Ertesvåg et al., 2009).

**FIGURE 3:**
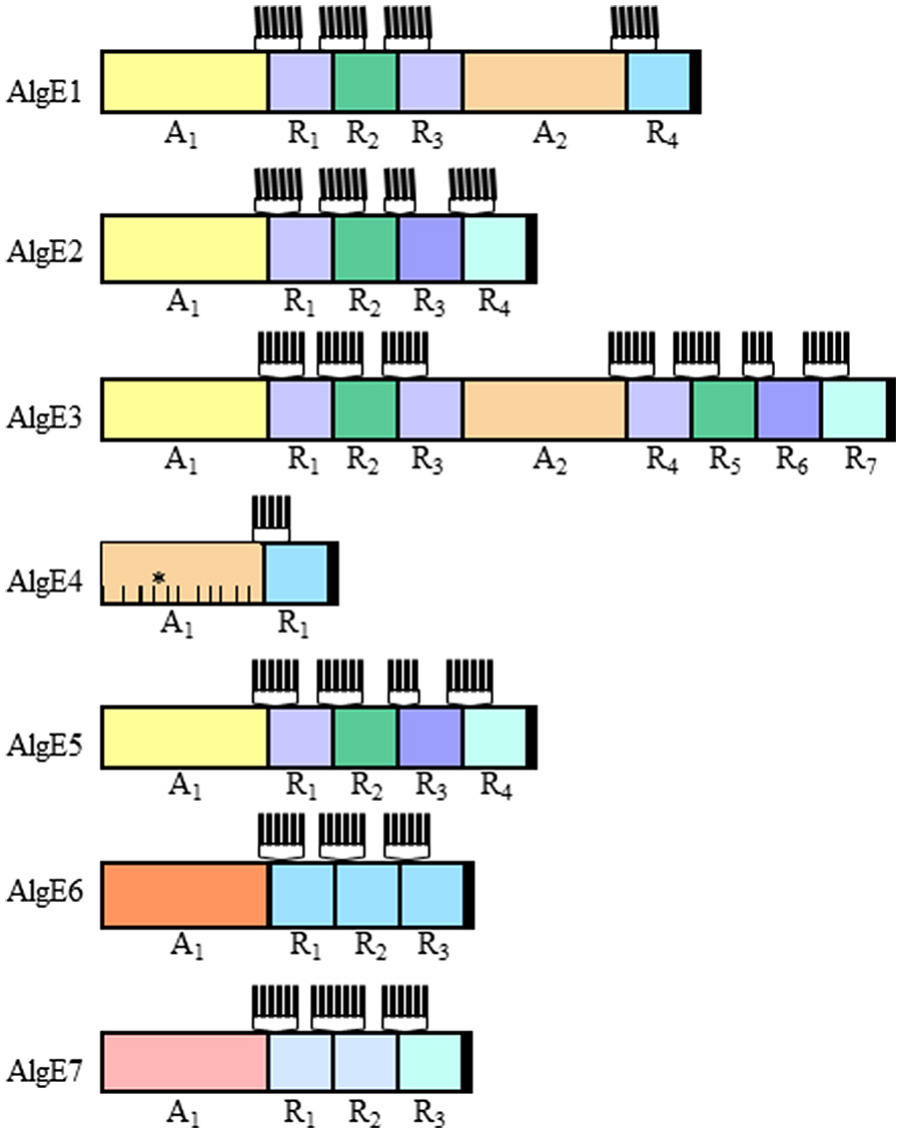
**The modular structure of the secreted epimerases from *Azotobacter vinelandii*.** The AlgE epimerases from *A. vinelandii* is composed of varying numbers of the two modules designated A and R. The relatedness between individual modules (confer **Figure 4**) is indicated by the similarity of the colors. The Ca^2+^-binding motifs found at the N-terminal end of each R-module are indicated by vertical lines. The proposed substrate-binding subsites in the A-module of AlgE4 are indicated by lines within the module, the catalytic subsite is marked with an asterisk.

The AlgE epimerases consist of two different protein modules where the A-module always is succeeded by from one to four R-modules (**Figure 3**). AlgE1 and AlgE3 each contain two A-modules. The A-modules are sufficient for both epimerization and for determining the epimerization pattern (Ertesvåg and Valla, 1999). The R-modules are needed for full activity (Ertesvåg and Valla, 1999) and has been shown to participate in substrate binding (Buchinger et al., 2014). At their N-terminal ends each R-module contain four to seven copies of a nine amino acid long motif known to be involved in binding of Ca^2+^ (Baumann et al., 1993; **Figure 3**).

**FIGURE 4:**
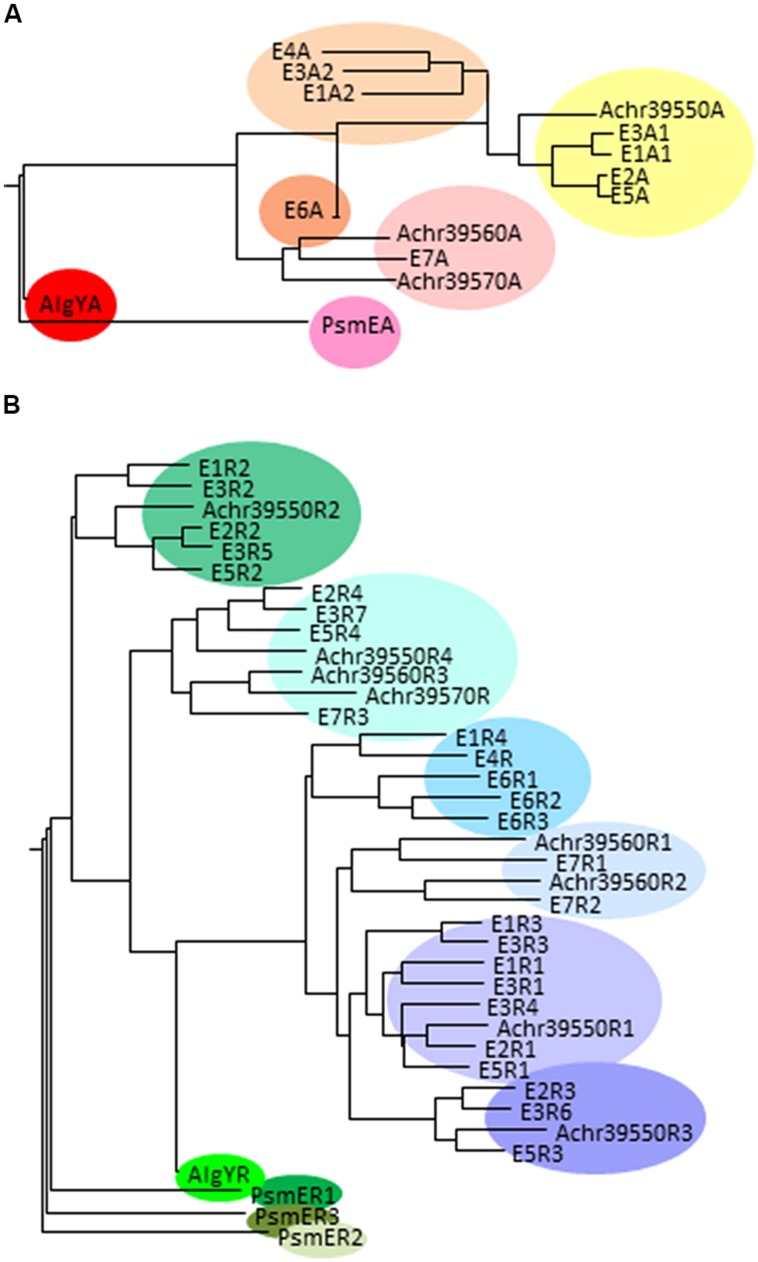
**Phylogenetic trees displaying the relationship between the A-modules **(A)** and R-modules **(B)** in different secreted mannuronan C-5 epimerases.** The *A. vinelandii* epimerases are denoted by E and enzyme number, AlgY is a protein homologous to AlgE-epimerases in *A. vinelandii*, PsmE is the *P. syringae* secreted epimerase, and the putative *A. chroococcum* epimerases are identified by their loci numbers. When one enzyme contains several A or R-modules, these are each numbered according to **Figure 3**.

The A-modules of the *A. vinelandii* enzymes differ when it comes to the G-distribution pattern they introduce in the alginate substrate. E1A2, E3A2, and E4A mainly introduce single G residues, while E1A1, E2A, E3A2, E5A, and E6A introduce consecutive G-residues as well as some single G residues. However, AlgE6A introduces longer G-blocks than the other A-modules. As mentioned above, AlgE7 is a bifunctional lyase/epimerase. It is able to introduce G-blocks, but also to cleave the alginate at an introduced G-residue. **Figure 4A** displays the similarity between the different A-modules showing that there is a clear relationship between the primary structure and the product. When it comes to the R-modules this is not the case; they tend to group mostly according to their position relative to the A-module (**Figure 4B**).

Six of the *A. vinelandii* AlgE epimerases are found clustered in the genome at around 5.2 Mb, while AlgE5 is found around 3.4 Mb. The *A. chroococcum* genome encodes only three putative secreted epimerases. These genes are found together in the same genomic context as the cluster containing six *A. vinelandii AlgE* genes. Both the A-module and the four R-modules of the protein encoded by *Achr_39550* are homologous to the corresponding modules of *A. vinelandii* AlgE2 (**Figure 4**). *Achr_39560* is transcribed in the same direction as *Achr_39550* and encodes a protein most similar to the bifunctional lyase/epimerase AlgE7. The third gene, *Achr_39570,* is transcribed in the opposite direction of *Achr_39560*. These two genes are fairly homologous except that 1058 nucleotides encoding amino acids 336-721 in *Achr_39560* is missing in *Achr_39570*. This truncated protein thus contain only nine of the 11 suggested subsites for substrate binding usually found in the A-module (Tøndervik et al., 2013), and only one R-module. Nothing is as yet known about the activities of these putative epimerases.

### AlgG and AlgE Epimerases Display Similar Structure and Catalytic Site Residues as Alginate Lyases

The structure of the E4A-module has been determined using x-ray crystallography (Rozeboom et al., 2008), while the structures of the R-modules of AlgE4 and of AlgE6 have been determined using NMR (Aachmann et al., 2006; Buchinger et al., 2014). These studies showed that both modules form right-handed β-helixes. A SAXS analysis of full length AlgE4 indicates that it forms a rod-shaped molecule (Buchinger et al., 2014). Both the A- and the R-modules participate in substrate binding, and it has been calculated that the A-module bind 11 uronic acid residues (**Figure 3**; Tøndervik et al., 2013), while the R-module can bind five residues (Buchinger et al., 2014). It has further been shown that different R-modules have different binding strength, and that this property influences the degree of processivity displayed by a given epimerase (Buchinger et al., 2014). The structure of *P. syringae* AlgG was recently published (Wolfram et al., 2014) and AlgG was found to form a long right-handed parallel β-helix similar to that of AlgE4. AlgG was calculated to bind at least nine uronic acid residues.

The catalytic mechanisms of lyases and epimerases were early postulated to be similar (Gacesa, 1987). Indeed, the close relationship between epimerases and lyases are emphasized by the bifunctional lyase/epimerase AlgE7 where the same catalytic site are needed for both activities (Svanem et al., 2001). Both epimerases and lyases need to neutralize the negative charge on the carboxyl group and to abstract the C-5-proton. For a lyase the last step then is cleavage of the glycosidic bond, while an epimerase needs an amino acid residue that is able to donate a proton to C-5 from the opposite side of the pyranose ring. The overall structure of both AlgG and the A-module of AlgE4 display structural similarities to some pectate- and pectin lyases of the PL1 family. While the negative charge in the Ca^2+^-dependent pectate lyases and AlgE4 is likely to be shielded by bound calcium, the calcium-independent pectin lyases, and AlgG use an arginine residue (Vitali et al., 1998; Wolfram et al., 2014).

The catalytic site of the epimerases otherwise seem to be more similar to that of alginate lyases from PL5 and PL7 (Rozeboom et al., 2008). Studies using AlgG mutants indicate that the histidine residue in the conserved DPHD motif found in both AlgG and the AlgE epimerases probably abstract the proton, while Tyr314 in AlgG coordinates a water molecule that might act as the proton donor (Wolfram et al., 2014). This tyrosine residue is also present in the catalytic site of AlgE4 (Rozeboom et al., 2008).

## Enzymes Involved in Determining the Degree of Acetylation

Alginate acetylation increases the water-binding capacity of the polymer (Skjåk-Bræk et al., 1989b). The acetyl groups will also increase the viscosity of the alginate and this might aid in keeping the polymer as a protective film close to the bacterium and hamper the movement of for instance immune cells. Acetylation also renders the mannuronic acid residue inaccessible for many alginate lyases, thus protecting the produced alginate from degradation (Skjåk-Bræk et al., 1989b). So far no studies have been published that compare the acetylation pattern for bacteria cultivated using different laboratory conditions or different strains.

### Four Proteins Encoded in the Alginate Biosynthetic Gene Cluster are Necessary for Alginate Acetylation

When the model describing the alginate polymerization and transport complex (**Figure 2**) were proposed a decade ago (See Alginate Lyases in Alginate-Producing Bacteria) it was known that AlgF, AlgI, and AlgJ were necessary for alginate acetylation, but also that none of these are necessary for polymer formation, indicating that they are not a part of the biosynthetic protein complex. The model could not explain how any of these enzymes could get access to the nascent alginate within the protein complex and acetylate it while AlgL could not degrade the polymer when it was surrounded by a functional complex. This enigma was recently resolved when it was shown that AlgX is the mannuronan *O*-acetylase (Riley et al., 2013; Baker et al., 2014).

AlgI is a membrane-bound *O*-acetyl-transferase that transfers an acetyl group from an unknown cytoplasmic acetyl donor (Franklin et al., 2004). The crystal structures of AlgX and AlgJ have been published and both enzymes belong to the SGNH hydrolase superfamily and further contain the same amino acids needed for *O*-acetyl-transferase activity (Riley et al., 2013; Baker et al., 2014). AlgJ is attached to the inner membrane (Franklin and Ohman, 2002), while AlgX is part of the alginate synthase, and transport complex. AlgX further contains a carbohydrate-binding module and has been shown to be able to acetylate mannuronan *in vitro* (Baker et al., 2014). AlgF is necessary for acetylation (Franklin and Ohman, 1993), however, it is not known which particular function AlgF has.

The alginate synthesis and export complex contains more proteins than for instance the cellulose synthesis and export complex encoded by *Gluconoactetobacter xylinus*, probably because alginate is modified during its passage through the periplasm (Whitney and Howell, 2013). *P. fluorescens,* which produces acetylated cellulose, encodes a modified cellulose synthase and export complex containing homologs of all four proteins necessary for alginate acetylation (Spiers et al., 2003).

It has been shown that bacterial alginates with a relatively high degree of acetylation has a lower degree of epimerization (Skjåk-Bræk et al., 1986). Given that the periplasmic epimerase AlgG and the acetylase AlgX are part of a protein complex, they may be considered as immobilized enzymes acting on a passing polymeric substrate. Both enzymes are able to bind several M-residues (Baker et al., 2014; Wolfram et al., 2014), and may exhibit some degree of processivity. It is not known if AlgG and AlgX are competing for binding to the alginate chain or if their potential access to any specific M-residue is separated in time.

### Alginate Deacetylation

Deacetylation may take place spontaneously, but the reaction is slow at normal pH. Extracellular epimerases are necessary for producing G-block containing alginate. However, these enzymes will not epimerize acetylated residues, and residues close to acetylated residues are probably also protected from epimerization. The crystal structure of AlgE4 indicates that this is due to steric constraints (Rozeboom et al., 2008). In this way the acetyl groups attached during the passage through the transport complex limit the amount of guluronic acid residues that can be formed later by the secreted epimerases.

So far no species of *Pseudomonas* have been found to produce alginate containing consecutive G-residues. However, *P. syringae* pv *glycinea* were found to a gene encoding a secreted mannuronan C-5-epimerase designated PsmE (Ullrich et al., 2000; Bjerkan et al., 2004a). PsmE was found to introduce consecutive G-residues *in vitro*. Similar to the secreted epimerases from *A. vinelandii,* PsmE contains a catalytic A-module and three Ca^2+^-binding R- modules. These all form a separate cluster in the phylogenetic trees (**Figure 4**). The enzyme contains two other Ca^2+^-binding modules, M and RTX and a seventh module called N containing 273 amino acid residues. In contrast to the secreted *A. vinelandii* epimerases, PsmE is able to epimerize acetylated substrates, and it was shown that the N-module is necessary for removing acetyl groups prior to epimerization by the A-module (Bjerkan et al., 2004a). Like AlgX and AlgJ, the N-module is predicted to belong to the SGNH hydrolase superfamily, and it shows some homology to esterases active on acetylated polysaccharides. Homologous genes are found in the genomes of many strains of *P. syringae* and related species (e.g., *P. savastanoi, P. amygdali, P. fluorescens,* and *P. avellanae*). Synthesis of bifunctional deacetylases/mannuronan C-5 epimerases would enable these strains to both decrease the amount of acetyl groups and to increase the G-content of its alginate.

## Use of Alginate Modifying Enzymes

For bulk applications such as food and feed, water treatment, or textile printing (Onsøyen, 1996) the alginate price may be as low as 5 USD per kg. However, alginate is also used in pharmaceutical and biotechnological industries, and for well-defined and ultrapure qualities the price may be as high as 100 USD/g. Examples of such applications are alginates for wound healing, encapsulation of pancreatic cells, and tissue engineering (Hunt and Grover, 2010; Skjåk-Bræk et al., 2015). The secreted mannuronan C-5 epimerases have been found to enable the upgrading of alginate *in vitro*, creating alginates with long G-blocks and/or replacing stretches of M-blocks with MG-blocks. The product may thus be tailored as to the need for gel strength, porosity, or biocompatibility (Skjåk-Bræk et al., 2015). It is also possible to tailor mannuronan using a chemoenzymatic approach in which some residues are modified and the material are then epimerized to introduce gel-forming G-blocks (Donati et al., 2005). In a recent paper this procedure was used to produce RGD-peptide modified alginates that were used to culture myoblasts in 2D and 3D cultures (Sandvig et al., 2015). Alternatively, specific epimerases might be used to obtain a homogenous population of alginate molecules with desired properties, which then are chemically modified (Arlov et al., 2014).

It is now possible to obtain pure mannuronan from bacteria synthesizing mutant AlgG proteins (Gimmestad et al., 2003). This may then be used as a substrate for recombinantly produced AlgE4, resulting in nearly pure MG-alginate (Holtan et al., 2006). G-blocks can be isolated from G-rich alginates from *L. digitata* (Haug et al., 1974). Such pure substrates are valuable when the substrate specificities of alginate lyases are to be determined.

Initial studies aimed at altering the substrate specificities of alginate lyases have been performed. The G-specific alginate lyase AlyA from *Klebsiella pneumonia* is able to degrade both G-M, and G-G bonds. The gene was subjected to random mutagenesis, cloned in a plasmid and transferred to *E. coli.* Protein extracts from 6700 randomly picked clones were prepared and assayed for lyase activity using isolated G-blocks and MG-alginate as substrates. One of the clones still retained sufficient activity on G-G bonds, while the activity on G-M-bonds was very low (Tøndervik et al., 2010). Recently it was also shown that the polysaccharide lyase Smlt1473 is active on several different polysaccharides, and, even more important, it was demonstrated that the activity and specificity of Smlt1473 toward polymannuronan and polyglucuronan could be increased by site-directed mutagenesis (MacDonald and Berger, 2014).

Alginate lyases are currently used to quantify alginate in a solution and may also be used to quantify the G-content (Østgaard, 1992). Still, as mentioned in Section “Post-Polymerization Modifications Determine the Functional Properties of Alginates” NMR is the most commonly used method to characterize alginate. However, NMR only yields knowledge of the statistical distribution of the M and G-residues. A new method utilizing a combination of alginate lyases specific for certain alginate bonds and HPAEC-PAD (Aarstad et al., 2012) enables a measurement of the block-length distribution to be performed, and this method has been used to show that algal alginates contain some very long G-blocks (Aarstad et al., 2013).

The homology and modular structure of the *A. vinelandii* AlgE epimerases has initiated several studies where modules or parts of modules are exchanged between the enzymes. Combining parts of the A-modules of AlgE2 and AlgE4 resulted in epimerases that introduced new G-distribution patterns (Bjerkan et al., 2004b). This approach was taken further when the DNA-fragments encoding the A-modules from AlgE1–AlgE6 was combined using error-prone PCR and cloned in front of the R-module from AlgE4. Nine hundred and sixty mutants from the resulting library were then screened for the ability to make long G-blocks and two mutant enzymes which met this criterion were identified (Tøndervik et al., 2013). This screen depended on the use of alginate lyases, included the GG-specific mutant lyase described above, to characterize alginate (Østgaard, 1992).

These studies illustrate that a random approach combined with an increased knowledge of the structure of the catalytic sites as well as the substrate binding sites of alginate lyases and epimerases will enable the design of new enzymes with more desired properties as to processivity, recognition site, substrate specificities, or catalytic activity. Engineered epimerases may then be used alone or in chemoenzymatic approaches to create biomaterials with new and desired functionalities.

## Conflict of Interest Statement

The author declares that the research was conducted in the absence of any commercial or financial relationships that could be construed as a potential conflict of interest.
